# Genetic and environmental contributions to the subjective burden of social isolation during the COVID-19 pandemic

**DOI:** 10.1186/s40359-023-01174-7

**Published:** 2023-04-26

**Authors:** Anita Kottwitz, Bastian Mönkediek, Christoph H. Klatzka, Anke Hufer-Thamm, Jannis Hildebrandt

**Affiliations:** 1 Faculty of Sociology, Bielefeld University, P. O. Box 10 01 31, 33501 Bielefeld, Germany; 2 Department of Psychology, Saarland University, Saarbruecken, Germany; 3 Department of Psychology, TU Dortmund University, Dortmund, Germany; 4 Faculty of Psychology and Sport Science, Bielefeld University, Bielefeld, Germany

**Keywords:** COVID-19 pandemic, Loneliness, Social isolation, Behavioral genetics

## Abstract

**Background:**

Feelings of loneliness and the burden of social isolation were among the most striking consequences of widespread containment measures, such as “social distancing”, during the COVID-19 pandemic. Because of the potential impact on people’s health, there has been increased interest in understanding the mechanisms and factors that contributed to feelings of loneliness and the burdens of social isolation. However, in this context, genetic predisposition has been largely ignored as an important factor. This is problematic because some of the phenotypic associations observed to date may in fact be genetic. The aim of this study is, therefore, to examine the genetic and environmental contributions to the burden of social isolation at two time points during the pandemic. In addition, we examine whether risk factors identified in previous studies explain genetic or environmental contributions to the burden of social isolation.

**Methods:**

The present study is based on a genetically sensitive design using data from the TwinLife panel study, which surveyed a large sample of adolescent and young adult twins during the first (N = 798) and the second (N = 2520) lockdown in Germany.

**Results:**

We find no substantive differences in genetic and environmental contributions to social isolation burden over the course of the pandemic. However, we find the determinants highlighted as important in previous studies can explain only a small proportion of the observed variance in the burden of social isolation and mainly explained genetic contributions.

**Conclusions:**

While some of the observed associations appear to be genetic, our findings underscore the need for further research, as the causes of individual differences in burden of social isolation remain unclear.

**Supplementary Information:**

The online version contains supplementary material available at 10.1186/s40359-023-01174-7.

## Introduction

Loneliness and burden of social isolation are known to be relevant and growing problems [1] that have significant health impacts on individuals and place a major burden on society's healthcare expenditures [2]. Often thought of as a problem for older people, younger age groups also feel lonely and socially isolated [3]. This also puts them at increased risk of future health problems, as loneliness is associated with physical and psychiatric disorders, including depression, sleep problems, personality disorders, and cardiovascular disease [4]. This risk has increased significantly, particularly in the context of the COVID-19 pandemic, where governments around the world have taken containment measures to reduce people's social contacts. As a result, broader segments of the population suddenly faced experiences of loneliness [5–9]. The measures mainly affected the daily lives of younger and middle-aged groups [10, 11]. However, not all adolescents and young adults seemed to be affected in the same way. For example, women in young adulthood had a higher incidence of loneliness than men in the same age range [12]. This makes it even more important to identify the groups that were more vulnerable to experience loneliness and social isolation in the course of the pandemic [13].

Several studies have identified factors that contribute to feelings of loneliness and social isolation, for example, age and gender [5–8, 11]. However, few studies examined the importance of these factors in the course of the pandemic [14, 15]. Even fewer studies considered the role of genetic predispositions [13]. This may be problematic because some of the previously observed associations may actually be genetic to some extent. For example, genetic predispositions that affect sociability could influence whether people live alone and how much they experience loneliness and social isolation during the pandemic. In such a case, the influence of living alone on feelings of loneliness and social isolation would be genetically confounded. Therefore, to understand individual differences in loneliness or the burden of social isolation and to identify vulnerable groups, a more in-depth study of factors is needed, which also considers genetic influences.

In this article, we use a genetically sensitive design to disentangle genetic and environmental contributions to burden of social isolation during the pandemic and examine any differences relative to previous research. We define subjective burden of social isolation (BSI) as the aversive state (i.e., feeling stressed or burdened) when the frequency or quality of social relationships is experienced as not satisfactory [16, 17]. We use this definition of BSI synonymously with the term loneliness (i.e., a person's subjective feeling that social relationships are perceived to be insufficient), as the distinction would lack discriminatory power, particularly during the pandemic (e.g., [18]). In addition, the definition does not include objective social isolation, which describes the actual quantity of social contacts [19].

The article is structured as follows. In the following chapter, we present the lockdown measures in Germany in the period from spring 2020 to July 2021, provide a brief insight into the current state of research and explain the objectives of this study in more detail. In the third chapter, we describe the study design and methods, followed by the results and a concluding discussion.

## Background

### Development of the pandemic and BSI in Germany

**Fig. 1:**
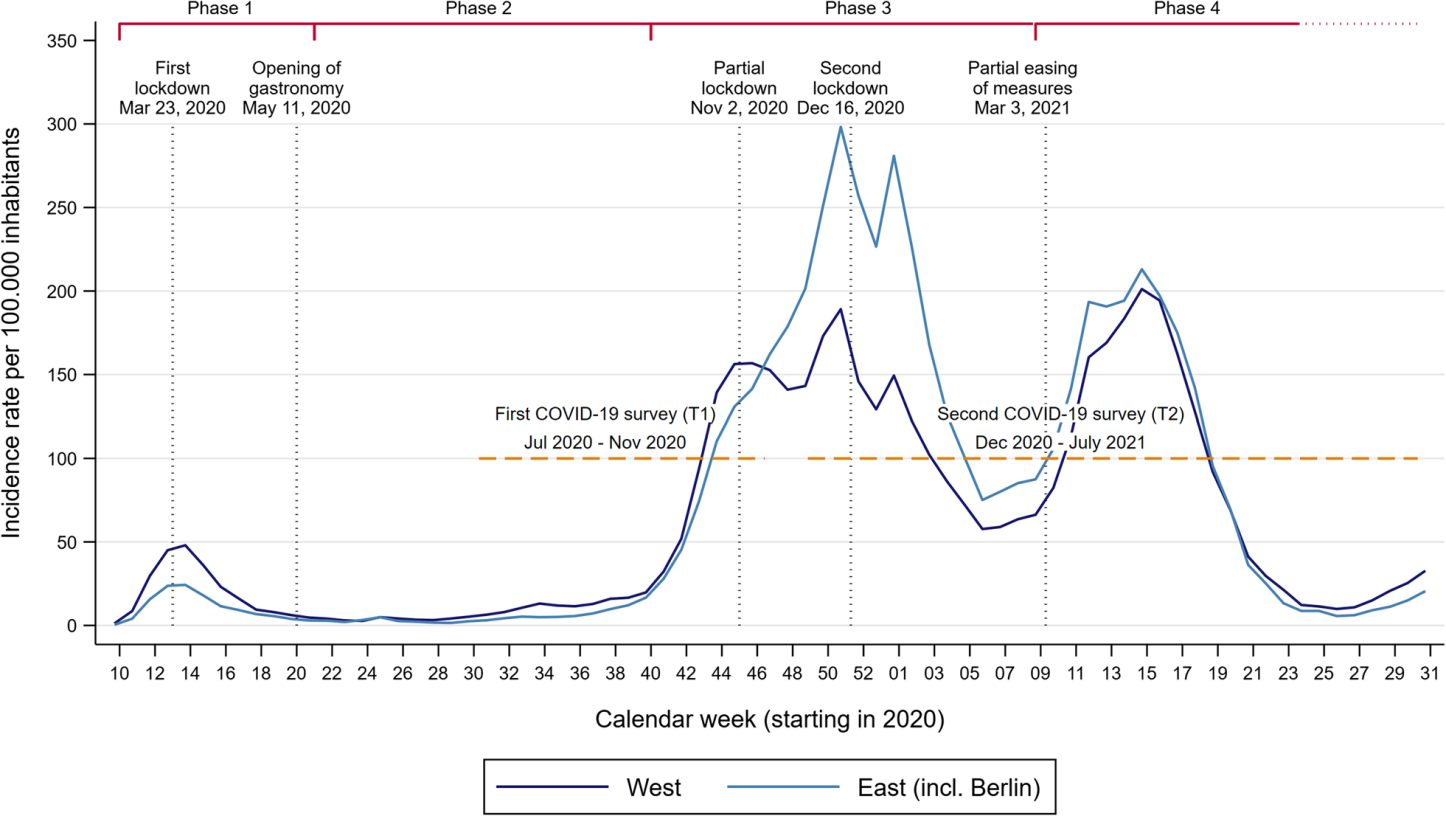
Development of the COVID-19 pandemic based on the incidence rate for East and West Germany in 2020 and 2021. First lockdown (Phase 1) = primarily contact restrictions in private life, travel restrictions, and closure of schools, gastronomy, retail, service sector, cultural institutions (e.g., museums, cinemas, concerts), and social sports facilities; Partial lockdown (Phase 2) = mainly contact restrictions in private life and closure of gastronomy, cultural institutions, and social sports facilities; Second Lockdown (Phase 3) = restrictions similar to first lockdown; Partial easing of measures (Phase 4) = gradual loosening of restrictions depending on the local incidence rate primarily regarding schools, gastronomy, retail, service sector, and cultural institutions. Source: [20, 22, 24, 25], own calculation

The COVID-19 pandemic began in Germany in March 2020 and developed in several phases [20–22] (Fig. 1). After the first wave of infections subsided after about three months because of a relatively strict lockdown, infection rates remained low in the summer of 2020 despite less restrictive measures (e.g., mandatory masks) [23]. The second, much more severe wave of infections began in the fall of 2020 and lasted until February 2021, transitioning into the third wave, which weakened in early summer 2021.

At the start date of the pandemic in 2020, studies reported only a slight increase in loneliness and no remarkably higher rates of mental health problems (e.g., [8]). Although loneliness was higher during the first two weeks of the nationwide lockdown (in March/April 2020), it appeared to have decreased in subsequent weeks [7]. However, a recent overview article pointed out that although most longitudinal studies found an increase in loneliness, most studies focused on comparing loneliness before the pandemic with loneliness during the pandemic and neglected changes during the pandemic [17]. More studies that consider multiple time points during the pandemic are therefore needed.

### Potential factors contributing to BSI

The pandemic situation may explain mean-level differences in BSI scores, i.e., whether BSI changed during the pandemic. However, as Rimfeld et al. [26] have elaborated, “this does not imply that differences in pandemic experiences are the sole source of individual differences in response to the crisis” (p. 111). Research has found several socio-demographic differences in the experience of BSI and loneliness during the pandemic that mirror the findings of pre-pandemic research (e.g., [5, 6, 27]). In a number of countries around the world, including Germany, women (e.g., [28]), individuals living alone [5, 6, 27], and people with low socioeconomic status (e.g., [27]) were observed to be more likely than others to report experiences of BSI and loneliness during the pandemic. The research also found new influencing factors, such as urbanity and age. Urban populations and young adults were found to be at higher risk of developing feelings of loneliness during the pandemic than people living in rural areas [6] and older people, respectively [29, 30]. Considering that BSI can be described as a mismatch between the need for social contact and the actual realized opportunities to socialize, these factors identified in previous research could influence either the need for social contact (such as age or gender) or the opportunities to socialize.

Humans typically differ in their genetic sensitivity to environmental conditions [31]. Therefore, groups that were more sensitive to changes in contact measures during the pandemic may be at higher risk for BSI. The recognition that people's responses to the same event may depend on their genetic predisposition [26] certainly makes genetic predispositions another important explanatory factor for individual differences in perceptions of the pandemic and its containment measures. Previous research explaining loneliness during the pandemic has largely ignored the fact that genetic influences contribute significantly to loneliness and BSI [31]. For example, Matthews et al. [32], using data from the mid-1990s, found that 40% of the variation in feelings of isolation and 38% of the variation in feelings of loneliness among young adults (aged 18 years) in the United Kingdom could be attributed to genetic variation. In addition, some of the phenotypic associations observed, such as the association between mental health problems and loneliness, may be due to genetic influences. For example, Freilich et al. [13] recently found that the association between certain personality dimensions and loneliness was largely due to shared genetic influences. Again, neglecting possible genetic influences may interfere with understanding the underlying mechanisms that contribute to loneliness experiences.

### The present study

Twin studies allow distinguishing between genetic, shared, and non-shared environmental influences on particular traits. Shared environment refers to the environmental factors that both twins are exposed to and experience in the same way. This usually includes factors like the home environment or parental socio-economic status. The non-shared environment refers to environmental factors that differ between the twins, such as distinct experiences or unique life events. In addition, the non-shared environment captures the importance of environmental factors that the twins objectively share but perceive differently. Given the need for further research that considers genetic influences, the present study uses a twin sample to examine the extent to which genetic, shared, and unshared environmental influences contributed to BSI during the first two waves of infection of the COVID-19 pandemic in Germany, which were characterized by severe restrictions such as lockdowns and other measures. In doing so, we extend previous research in two ways:

First, we consider genetic predispositions as another important explanatory factor for differences in BSI and recognize that people may have responded differently to containment measures because of genetic predispositions that influence their sensitivity to the environment. In other words, we address the question: (Q1) To what extent did genetic predispositions contribute to BSI during the pandemic? To our knowledge, this is the first study to examine BSI during the COVID-19 pandemic using a genetically sensitive design. Second, although genetic and environmental contributions to BSI may have changed during the course of the pandemic, it is not clear to date whether and to what extent such changes occurred. The few existing genetically sensitive studies known to the authors examined changes in genetic and environmental contributions to various psychopathological traits, including general anxiety and depression, but not BSI [26]. Therefore, our second question is: (Q2) Did the pattern of genetic, shared, and non-shared environmental contribution to BSI change throughout the course of the pandemic?

Third, by combining a phenotypic and a behavioral genetic approach, we aim to examine whether previously observed associations were driven by underlying genetic influences (Q3). While it is nearly impossible for a single study to account for all potential factors influencing BSI—there is always a risk of bias from omitted variables—, our variance decomposition models allow to more comprehensively control for factors that have not been directly measured [33].

## Materials and methods

### Data collection

Data came from the TwinLife project, a cohort-sequential longitudinal study of initially over 4000 same-sex monozygotic (MZ) and dizygotic (DZ) twins from Germany who grew up together, started in 2014 [34, 35]. The TwinLife study received ethical approval from the German Psychological Society (Deutsche Gesellschaft für Psychologie; protocol number: RR 11.2009) and thus met the ethical standards of the 1964 Helsinki Declaration and its subsequent amendments. The Corona supplemental survey was also approved as ethically safe by the Ethics Committee of the University of Bielefeld (Application No. 2020-106). Prior to the interview, participants were comprehensively informed in writing about the scope and purpose of the study, their right to refuse or withdraw from participation at any time, and the data protection regulations. Informed consent was obtained from all participants and their legal guardians if they were under 14 years of age.

This multidisciplinary study aims to examine the genetic and environmental influences that shape social inequalities across the life course. TwinLife includes four birth cohorts of twins: the youngest twins in Cohort 1 were born in 2009 or 2010, the twins in Cohort 2 were born in 2003 or 2004, the twins in Cohort 3 were born in 1997 or 1998, and the oldest twins in Cohort 4 were born between 1990 and 1993. The 2009, 2003, 1997, and 1990–1991 birth cohorts from subsample A were first interviewed in 2014. The 2010, 2004, 1998, and 1992–1993 birth cohorts from subsample B were included in the TwinLife panel in 2015. Thus, these twins were approximately 5, 11, 17, and 23 to 24 years old at the time of the first panel wave. Families are contacted annually, starting with a face-to-face interview followed by a telephone interview the following year. In subsequent years, this alternation between survey modes has continued. The TwinLife sample is comparable to families with two or more children of the same birth cohorts from the German Microcensus, which is based on a sample that is representative for Germany [36]. This applies, for example, to the mother’s age at birth, the proportion of single-parent households, municipality size classes of the place of residence, and socio-economic characteristics. However, parents with a higher level of education are slightly overrepresented, and those with a migration background are slightly underrepresented in the TwinLife sample, which may be due to the fact that extensive questionnaire programmes can be a barrier for these families [36].

After the start of the COVID-19 pandemic in Germany, additional COVID-19 surveys were added to the TwinLife study, and in some cases, an additional module was integrated into the regular surveys. The data from these supplemental surveys will be used to investigate our research questions. The first supplemental COVID-19 online survey (T1) started in late July 2020 and ended in early November 2020, with August 2020 as the median survey month. The survey was conducted at a time when there was no longer a lockdown in Germany and incidence rates were relatively low, but the content of the assessments *retrospectively* referred to the first lockdown period in Germany that began in March 2020 (Fig. 1). A total of 399 complete MZ and DZ twin pairs from birth cohorts 2, 3, and 4 (2003/2004, 1997/1998, and 1990–1993) who were at least 16 years old at the start date of the survey were selected from the sample. Twins younger than 16 were asked different questions in the subject area studied, so they were not included in this analysis. The final sample included 798 individuals, with mean ages of 16.4 (SD = 0.5), 22.1 (SD = 0.7), and 28.3 (SD = 1.1) years in cohorts 2, 3, and 4, respectively. The sample was composed of 194 MZ twin pairs and 205 same-sex DZ twin pairs.

The second COVID-19 survey (T2) was conducted between December 2020 and April 2021 (subsample A) and from February 2021 to July 2021 (subsample B) as part of the regular data collection of TwinLife. T2 serves as the data basis for the second and third infection wave of the pandemic. Interviews were conducted over the phone by the interviewer from the previous face-to-face survey waves. In contrast to the first data assessment, this survey referred to the *current* situation of the respondents and was started at the beginning of the second lockdown in Germany (Fig. 1). In total, the second survey included 2520 individuals (1260 twin pairs) with mean ages of 16.6 (SD = 0.5), 22.6 (SD = 0.5), and 28.7 (SD = 1.1) years in cohorts 2, 3, and 4, respectively. The sample was composed of 670 MZ twin pairs and 590 same-sex DZ twin pairs.

### Measures

#### Dependent variables

To measure the experienced BSI as a result of the first phase of the pandemic (T1), participants were asked, “Thinking […] about the first period of the Corona Crisis, i.e., from March 2020 until the first ease of restrictions, how much did you feel the following burden?” The specific burdens were “social isolation/loneliness,” “being separated from important people,” and the “lack of leisure activities” (adapted from [37]). The wording of the questions for the second COVID-19 survey (T2) was slightly adjusted to read, “The Corona pandemic continues to affect the daily lives of many people. To the extent that you are currently experiencing the following limitations, how much do you feel the following burden?” In both COVID-19 data collections, respondents could rate their answers on a Likert scale from 1 “No burden” to 10 “Extreme burden” for each of the three items. The response “Does not apply” was recoded to 1. The mean score of these three items was calculated and z-standardized (M = 0; SD = 1).

A principal-component factor analysis with oblique factor rotation [38] revealed a common factor for the three items on BSI due to the Corona pandemic, with factor loadings ranging from 0.696 to 0.829 (Additional file 1: Table S1). The average inter-item correlations as an indicator of unidimensionality were *r* = *0*.399 (T1) and *r* = *0*.432 (T2), which were within the ideal range of 0.15–0.50 [39]. Both reliability measures for the three-item scale of BSI, Cronbach's alpha (0.666 in T1 and 0.695 in T2) and McDonald's omega (0.680 in T1 and 0.719 in T2) are slightly below or above the recommended minimum of 0.70 [40, 41] However, bearing in mind that shorter scales and smaller sample sizes tend to have lower Cronbach's alpha values, we consider the calculated coefficients to be acceptable for the purposes of our study.

#### Covariates

We measured pre-pandemic levels of loneliness to determine the extent to which prior loneliness experiences influenced BSI during the pandemic. *Loneliness before first lockdown* was assessed using a paper–pencil questionnaire asking “To what extent do you agree with the following statement? I often feel lonely” on a Likert scale from 1 “totally disagree” to 4 “totally agree.” Loneliness was assessed during the first, second, and third waves of the TwinLife study, which took place between September 2014 and April 2016, November 2016 and May 2018, and November 2018 and June 2020, respectively. We calculated the mean score of the three previous loneliness ratings during the TwinLife face-to-face panel waves and z-standardized (*M* = *0*; *SD* = *1*) the resulting new variable. Loneliness ratings in the third face-to-face survey received after March 15, 2020, were not included in the calculation of the mean due to the overlap with the first corona-related lockdown in Germany.

Previous studies have shown that urban populations appeared to be at higher risk of loneliness during the pandemic than people living in rural areas [6]. *Urban–rural differences* were controlled for using available information on the population size of respondents' places of residence. The categorization was based on the German spatial classification system BIK [42]. This variable was dummy-coded and included whether the participant lived in a core area with more than 50,000 inhabitants or not.

There were regional differences in incidence rates [43] and containment measures taken among regions in Germany, particularly between eastern and western Germany (see Fig. 1). These differences likely contributed to how the pandemic situation was experienced. We examined the extent to which this was the case by including a dichotomous variable for place of residence in eastern Germany.

Using information on household size, we study the extent to which individuals living in a *single household* report higher levels of BSI than all others. We do not have a measure to control for objective social isolation for the measurement points. Instead, we used “living alone” as an alternative measure as household size has been identified as an important factor in explaining aspects of BSI. Research has shown that individuals living alone, in particular, are at higher risk of reporting loneliness [6, 27]. The covariate *single household* cannot directly measure objective social isolation, as a person can also have social contacts outside the household. However, we assume that due to “social distancing” measures during the lockdowns, “objective social isolation” has been more homogeneous in the general population than at any other measurement points before the pandemic, minimizing the risk of bias. Also, some studies have shown that objective social isolation and loneliness are related but only weakly correlated overall [44, 45].

The relationship between BSI and socioeconomic factors has not always been clear in previous research, partly due to different operationalizations. Previous studies have found a negative or no relationship between BSI and socioeconomic factors such as education or income. This is true for surveys conducted before the pandemic as well as those conducted during the pandemic (for a literature review, see [46]). In our study, we considered *economic position* and *educational level* as determinants of BSI. To measure *economic position*, the equivalent income based on the modified OECD equivalence scale was divided into four categories based on the median of the net equivalent income (NEI) of the population in Germany in 2019 [47]: (1) “Risk of poverty (< = 60% of NEI) and low-income middle class (> 60–80% of NEI)” as reference category, (2) “middle class (> 80–120% of NEI)”, (3) “upper and high-income class (> 120% of NEI)”, and (4) “missing information on income”. The *educational level* was formed using the highest years of education of the mother of the twins.

Since regional information (BIK, East), household size, and household income were not collected for T1, information from the late 2019/early 2020 survey was used for the analysis of T1.

#### Control variables

We controlled for *birth cohorts* and *sex* of twins in our analyses because age and sex might artificially increase the intraclass correlation of twins and thus lead to an underestimation of heritability [48]. In addition, studies have shown that women were more likely to feel lonely during the pandemic (e.g., [12]). Because data collection was conducted over a period of several months, we also controlled for the *survey month* since the start date of the field period (T1: July 2020, T2: December 2020) to account for changes in the pandemic situation and its impact on BSI experiences on the one hand and respondents’ motivation to participate on the other.

### Analytic strategy

Our analysis combined a phenotypic with a behavioral genetic approach. A brief introduction to the concepts and methods of behavioral genetics, including an overview of different types of twin models, their applications, advantages, and limitations, is given by [49]. Twin-based genetic studies use the genetic relatedness of monozygotic (MZ; 100% genetic similarity) and dizygotic (DZ) twins (who share on average 50% of their segregating genes) to decompose the observed variance of a trait, such as BSI, into typically three additive, latent variance components (see Box 1): a genetic component (A), a shared environmental component (C), and a non-shared environmental component (E). In other words, the observed variance in a trait is captured and partitioned among the latent components. In this context, A refers to the extent of phenotypic trait variation in a population that is due to genetic variation, i.e., heritability [50, 51]. As discussed by Freese [52], genetic variation influences traits through embodied characteristics. Therefore, A can be considered as a placeholder for all types of genetically influenced traits, such as physical or psychological traits that have not been directly measured. C refers to all environmental influences that twins share, such as family environment or neighborhood, that increase the correlation of twins in a trait [51]. However, not all objectively shared environments of twins need to have uniform effects on them. For example, family environment or parenting may be perceived differently by twins in the same family. Similarly, growing up in a small village may be perceived as positive by one twin, which could reduce BSI, for example, and as very negative by the other twin, which could increase BSI. In both cases, these influences will enter the E component, the non-shared environment. In addition to such differential experiences of objectively shared environments, E additionally refers to any unique experiences of the twins that reinforce their trait differences [51, 53].

**Box 1:** Definitions (see [50, 51])

*Heritability* refers to the extent of phenotypic trait variation in a population that is due to genetic variation
*The genetic component (A)* refers to narrow sense heritability, which describes the proportion of the variance of a trait that is explained by additive genetic effects
*The shared environment component (C)* refers to influences related to the common environment of twins that increase the correlation of twins in a trait
*The non-shared environment component (E)* refers to influences related either to the twins’ shared environment that are experienced differently, or to specific and incidental experiences of the twins that reinforce the twins' dissimilarity in a trait

**Fig. 2:**
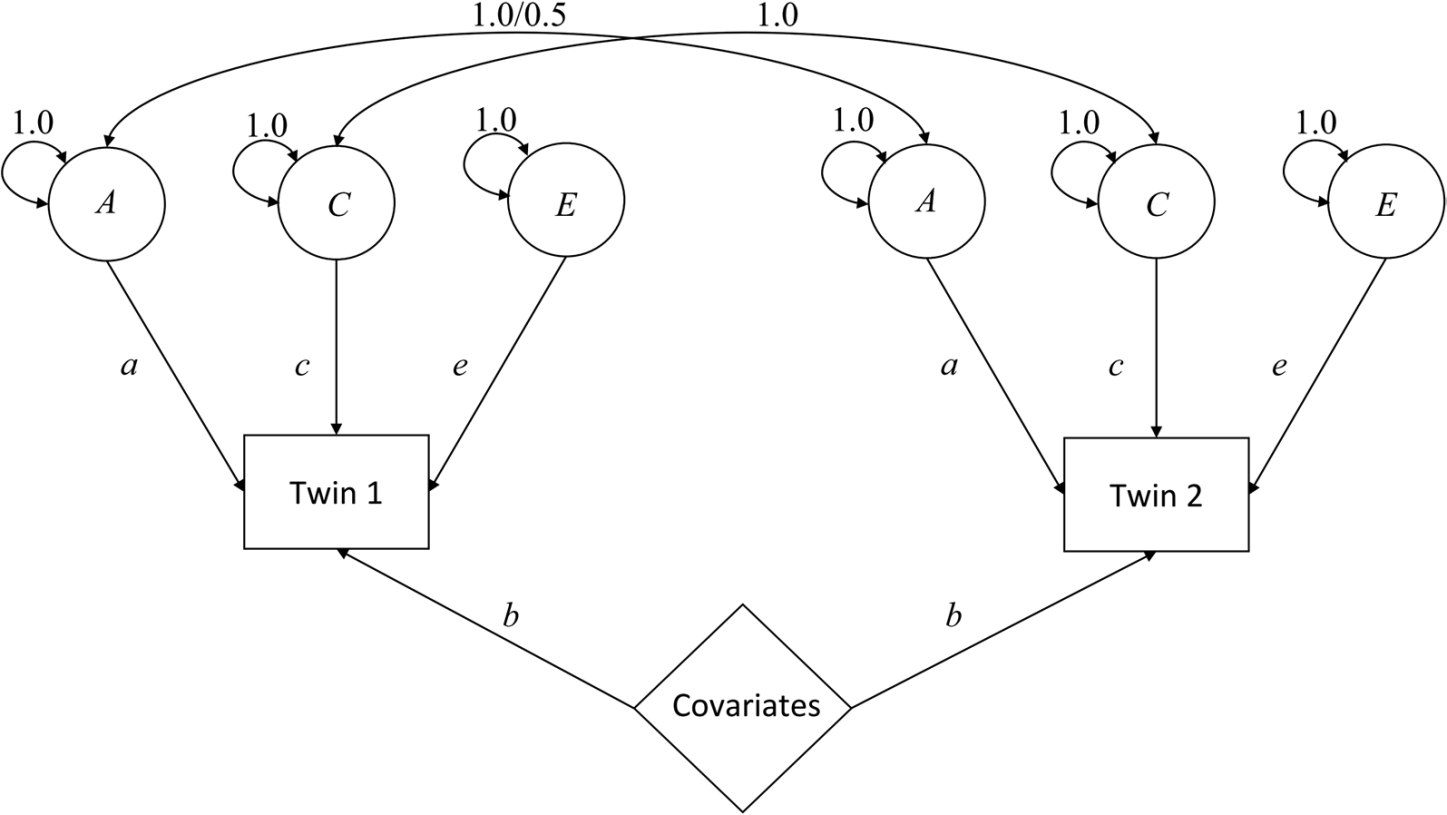
Univariate ACE path diagram with covariate effects. Note: The variance components of the latent variables A, C, and E are fixed at the value 1 so that the path coefficients a, c, and e are estimated. The 1.0/0.5 covariance link between the two latent A variables represents the unequal genetic relatedness between MZ and DZ twins: for MZ twins, this covariance is fixed at 1; for DZ twins, it is fixed at 0.5. b are the phenotypic means/intercepts of the covariates

The variance components, as latent random variables, are typically estimated in path analysis (Fig. 2; for an introduction, see [54]).

In the path model, the observed characteristics of twin 1 and twin 2 are postulated to depend on these three latent random variables (A, C, E) and the model means [55]. It is assumed that components A, C, and E are additive and that there are no correlations or interactions between them [55, 56]. The single-track arrows in Fig. 2 denote the postulated causal path, and the small letters (a, c, e) denote the estimated effect sizes [55]. According to the rules of path tracing [57], the covariance structure of a trait can be derived (1). For example, genetic covariance can be inferred by tracing the genetic path from twin 1 to twin 2. For MZ twins, this results in: a*1*a = a^2^, and for DZ twins: a*0.5*a = 0.5a^2^. Similarly, the covariance related to shared environmental influences is calculated as follows: c*1*c = c^2^ (for both MZ and DZ twins).

1 $$\begin{gathered} {\text{Cov}}_{MZ} = \left( {\begin{array}{*{20}c} {a^{2} + c^{2} + e^{2} } & {a^{2} + c^{2} } \\ {a^{2} + c^{2} } & {a^{2} + c^{2} + e^{2} } \\ \end{array} } \right) \hfill \\ {\text{Cov}}_{DZ} = \left( {\begin{array}{*{20}c} {a^{2} + c^{2} + e^{2} } & {0.5a^{2} + c^{2} } \\ {0.5a^{2} + c^{2} } & {a^{2} + c^{2} + e^{2} } \\ \end{array} } \right) \hfill \\ \end{gathered}$$

2 $$rMZ = \frac{{a^{2} + c^{2} }}{{\left( {a^{2} + c^{2} + e^{2} } \right)}}\quad rDZ = \frac{{0.5a^{2} + c^{2} }}{{\left( {a^{2} + c^{2} + e^{2} } \right)}}$$

3 $${\text{A}} = 2 * \left( {{\text{rMZ}} - {\text{rDZ}}} \right)$$

4 $${\text{C}} = 2 * {\text{rDZ}} - {\text{rMZ}}$$

5 $${\text{E}} = 1 - {\text{rMZ}}$$

Based on the observed covariance structure (1) between and within MZ and DZ twin pairs, the variance components can be calculated (2–5). Under the equal environment assumption (EEA) between MZ and DZ twins, the heritability of BSI (A) is calculated as twice the differences of the MZ correlation (rMZ) minus the DZ correlation (rDZ) (3). Previous studies have shown that even when the EEA is not met, this is usually unproblematic at the analytic level [53, 56]. C is obtained by subtracting the heritability coefficient from rMZ, and E is obtained by subtracting rMZ from one [56] (5).

We tested the ACE model against other model variants that either omitted the additive genetic component (CE) or the shared environmental effect (AE). Here, we selected the most parsimonious model, which didn’t significantly underfit the data, as indicated by the p-values of the likelihood-ratio test [58]. We also compared the Akaike information criterion (AIC) and the Bayesian information criterion (BIC) for the different models [59, 60].

We conducted our analysis in two steps. In the first step, we examined the phenotypic effects of measures identified in previous research as related to BSI using a univariate ACE model with covariates (Fig. 2). Like our control variables, the covariates also entered the means model and thus captured part of the variance of the BSI. The coefficients for the covariates and the control variables are consistent with a typical regression analysis and can be interpreted in the same manner. A negative sign of the coefficients indicates a decrease in BSI, and a positive sign indicates an increase in BSI.

In the second step, we focused on the results of the behavioral genetics part of our analyses, which we report separately. Here, we examined how the magnitude of the variance components A, C, and E changed when covariates were included. To do this, we compared the variance components for a “reduced model,” in which we controlled only for birth cohorts and sex, and a “full model,” in which we included all covariates for which we expected an effect on BSI. The reduced model allowed us to determine how genetic, shared, and unshared environmental influences contributed to BSI during the pandemic. The full model only decomposed the remaining variance that could not be explained by our covariates and control variables and assigned it to the variance components. By comparing the estimates across models, we were able to assess the extent to which our measured variables carried genetic and environmental influences [61]. To this end, we divided the difference between the standardized coefficients of the reduced and full models by the total variance of the reduced model ((a^2^, c^2^, e^2^_reduced model_ − a^2^, c^2^, e^2^_full model_)/(a^2^ + c^2^ + e^2^) _reduced model_). Finally, we examined the variance components (A, C, E) for two time points during the pandemic to determine whether the context of the pandemic situation might have influenced the contributions of genetic and environmental influences to BSI. For robustness, we additionally performed the analyses for T1 and T2 for the sample that participated in both surveys (N = 722). Behavioral genetic analysis was run in R using the package umx, version 4-9-0 [62].

## Results

The descriptive statistics for BSI are presented in Table 1. At T1, BSI averaged 4.584 (SD = 2.150) on a scale of 1 (no burden) to 10 (extreme burden). At T2, BSI increased significantly, averaging 5.345 with a lower standard deviation of 2.039 (Cohen’s d = 0.368).

**Table 1:** Descriptive statistics

	T1: 2020		T2: 2021	
	M	SD	M	SD
Burden: social isolation/loneliness	3.995	2.695	4.583	2.503
Burden: being separated from important people	4.737	2.783	5.307	2.545
Burden: lack of leisure activities	5.020	2.861	6.146	2.732
Burden of social isolation (3-item-score)	4.584	2.150	5.345	2.039
Loneliness (mean of up to 3 measurements before first lockdown)	1.715	0.635	1.738	0.659
Observations	798		2520	
N twin pairs	399		1260	

*Source*: TwinLife CoV1 v6-0-0 (T1), CoV2 (T2); Sample: cohorts 2, 3 & 4

Descriptive statistics for the covariates between the two data collections showed no or only moderate differences (Table 2).

**Table 2:** Sample description

	T1: 2020		T2: 2021	
	M/%	SD	M/%	SD
Zygosity: monozygotic	49%		47%	
*Birth cohorts*				
Cohort 2: 2003/2004	46%		45%	
Cohort 3: 1997/1998	30%		31%	
Cohort 4: 1990–1993	24%		24%	
Gender: female	56%		55%	
Spatial area: > = 50.000 core area	68%		70%	
Eastern federal states	18%		17%	
Single household	12%		18%	
Highest educational level of mother of twins	15.153	2.190	14.928	2.401
*Income position of household*				
Risk of poverty/low-income middle class (< = 80% of median of net equivalent income)	31%		33%	
Middle class (> 80–120%)	34%		31%	
Upper/high-income (> 120%)	24%		30%	
Information on income is missing	11%		6%	
TwinLife subsample A	67%		54%	
Month of interview since start of data collection	2.089	1.145	2.887	1.699
Observations	798		2520	
N twin pairs	399		1260	

*Source*: TwinLife CoV1 v6-0–0 (T1), CoV2 (T2); Sample: cohorts 2, 3 & 4

BSI was higher in 22- and 23-year-old twins (cohort 3) than in 16-year-old twins (cohort 2) at T1 in spring 2020 (Table 3, column 1). Female twins also exhibited increased BSI scores despite controlling for pre-pandemic loneliness experience. Individuals who had experienced more loneliness before the pandemic felt more distress from social isolation during the first lockdown, i.e., a one standard deviation increase in loneliness before the pandemic led to a 0.117 standard deviation increase in BSI during the first lockdown. Resources such as education or income and living in a single household did not affect BSI during the first lockdown.

**Table 3:** Effects of covariates on burden of social isolation (T1, T2)

	T1: 2020				T2: 2021			
	Reduced model		Full model		Reduced model		Full model	
	Coef.	95% CI	Coef.	95% CI	Coef.	95% CI	Coef.	95% CI
Cohort 2: 2003/2004 (ref.)	Ref		Ref		Ref		Ref	
Cohort 3: 1997/1998	0.319*	[0.140,0.499]	0.267*	[0.081,0.453]	0.366*	[0.266,0.466]	0.310*	[0.204,0.416]
Cohort 4: 1990–1993	0.239*	[0.045,0.432]	0.211	[− 0.004,0.426]	0.316*	[0.207,0.426]	0.257*	[0.135,0.380]
Gender: female	0.311*	[0.156,0.466]	0.285*	[0.132,0.437]	0.310*	[0.223,0.396]	0.294*	[0.207,0.381]
Spatial area: > = 50.000 core area			− 0.009	[− 0.168,0.151]			0.048	[− 0.045,0.140]
Eastern federal states			− 0.155	[− 0.353,0.043]			0.053	[− 0.059,0.164]
Single household			0.127	[− 0.100,0.355]			0.108	[− 0.003,0.219]
Highest educational level of mother of twins			0.030	[− 0.007,0.066]			− 0.001	[− 0.020,0.018]
Risk of poverty/low-income middle class (< = 80% of median of net equivalent income) (ref.)			Ref				Ref	
Middle class (> 80–120%)			0.035	[− 0.150,0.219]			0.045	[− 0.057,0.148]
Upper/high-income (> 120%)			0.059	[− 0.153,0.271]			0.011	[− 0.098,0.119]
Information on income is missing			0.139	[− 0.113,0.390]			0.022	[− 0.153,0.197]
Loneliness before first lockdown			0.117*	[0.048,0.186]			0.070*	[0.031,0.108]
Intercept	− 0.324	[− 0.462,− 0.185]	− 0.948	[− 1.554,− 0.342]	− 0.359	[− 0.436,− 0.282]	− 0.447	[− 0.802,− 0.093]
N	798		798		2520		2520	
N twin pairs	399		399		1260		1260	

*Source*: TwinLife v6-0–0 CoV1 (T1), CoV2 (T2); Sample: cohorts 2, 3 & 4

Further control variables: sub-sample A, month of interview since start of data collection

*p < 0.05

We found similar associations in the second measurement starting in winter 2020/2021 (Table 3, column 2): Women and 23-year-old twins (cohort 3) had higher BSI scores compared with men and the other birth cohorts, respectively. Individuals living alone did not have higher BSI scores compared with individuals living in multi-person households at both measurement time points T1 and T2. Interestingly, there were also no differences between people with lower and higher incomes. Accordingly, not all observed associations between covariates and BSI pointed in the expected direction that we had inferred from previous studies.

The behavioral genetic variance decomposition of the final models is shown in Table 4. For this, we compared the full ACE model (Additional file 1: Table S2) with its nested AE and CE models. Based on the Akaike information criterion (AIC), Bayesian information criterion (BIC), and a likelihood ratio test, the best-fitting models were an AE model for T1 (i.e., containing only additive genetic and non-shared environmental factors) and an AE model for T2, as shown in Additional file 1: Table S3. In other words, for both measurement time points, the best fitting models indicate no significant contributions of shared environmental influences on BSI for the twins. This result is consistent with our finding that there is no influence of covariates that are most likely to capture such shared environmental influences on BSI, such as state, maternal education, or household income.

**Table 4:** ACE decomposition of burden of social isolation (T1, T2)

	T1: 2020				T2: 2021			
	Reduced model		Full model		Reduced model		Full model	
*Twin correlations*								
MZr	0.370	[0.236,0.505]	0.334	[0.197,0.471]	0.392	[0.317,0.466]	0.362	[0.286,0.437]
DZr	0.250	[0.120,0.380]	0.219	[0.082,0.357]	0.261	[0.191,0.331]	0.243	[0.170,0.315]
*ACE decomposition (unstandardized variance components)*								
a^2^	0.348	[0.237,0.467]	0.321	[0.212,0.437]	0.366	[0.302,0.432]	0.355	[0.292,0.421]
c^2^								
e^2^	0.603	[0.510,0.716]	0.597	[0.504,0.710]	0.575	[0.522,0.635]	0.575	[0.522,0.635]
Total variance (a^2^ + c^2^ + e^2^) (residual variance)	0.951		0.918		0.941		0.930	
Variance explained^a^			3.5%				1.2%	
*ACE decomposition (standardized variance components)*								
A	0.366	[0.256,0.465]	0.350	[0.237,0.452]	0.389	[0.327,0.446]	0.382	[0.320,0.440]
C								
E	0.634	[0.535,0.744]	0.650	[0.548,0.763]	0.611	[0.554,0.673]	0.618	[0.560,0.680]
A + C + E	1.000		1.000		1.000		1.000	
N	798		798		2520		2520	
N twin pairs	399		399		1260		1260	

*Source*: TwinLife v6-0-0 CoV1 (T1), CoV2 (T2); Sample: cohorts 2, 3 & 4

Reduced model adjusted for birth cohort and sex; 95% CI in brackets

^a^The proportion of variance explained by full model is calculated as follows: (variance _reduced_ − variance _full_)/variance _reduced_

At T1, in the reduced model, 37% of the variance in BSI was due to genetic influences, and 63% of the variance was due to non-shared environmental influences. In this analysis, adding the covariates to the model explained 3.5% of the differences between twins, whereas the remaining variance was explained by genetic influences (35%) and non-shared environmental influences (65%). The only covariate with significant influence to which the explained variance can be attributed is the experience of loneliness before the pandemic. Interestingly, more than three-quarters of this explained variance ((0.348 − 0.321)/0.951 = 2.8%) was attributed to the genetic component in the reduced model. The remainder of the explained variance belongs to the effects of unsystematic experience (E). Thus, pre-pandemic loneliness experiences seem to influence BSI during the pandemic due to genetically transmitted influences and existing individual experiences.

We found similar results at T2 in both the reduced model and the full model. Genetic (reduced model: 39%; full model: 38%) and non-shared environmental (reduced model: 61%; full model: 62%) influences continued to play an important role. Inclusion of covariates in the model explained only 1.2% of the variance in BSI ((0.366 − 0.355)/0.941 = 1.2%). All explained variance was attributed to the genetic component in the reduced model. All results were robust to analyses with twin pairs (N = 722) who participated in both surveys T1 and T2 (results can be provided by the authors on request).

## Discussion

Numerous studies analyzed the psychosocial consequences of COVID-19 containment measures, including social distancing, e.g., on the subjective burden of social isolation (BSI) (e.g., [63]). In this context, previous research shows that a variety of factors influence BSI at different levels (at the individual level, at the family and household level, and at the local and regional level) [5, 6, 27, 29, 30]. However, genetic influences have often been ignored as important explanatory factors. While people’s individual responses to the pandemic and the containment measures may be influenced by their genetic makeup [26], some of the phenotypic associations observed previously may be due in part to genetic confounding [13]. Addressing this issue, the present article used a twin-based approach and investigated the extent to which genetic and environmental influences contributed to BSI during the first two infection waves of the COVID-19 pandemic in Germany. Based on the applied approach, we additionally considered the possibility that unobserved individual characteristics and unobserved environmental factors influenced BSI during the pandemic. To the authors' knowledge, this study was the first to examine BSI associated with the COVID-19 pandemic in Germany using a genetically sensitive design.

In answer to our first research question (Q1), namely, to what extent genetic predispositions contributed to BSI during the pandemic, our results suggested that genetic predisposition and non-shared environmental factors affected lockdown-related BSI. In contrast, shared environmental factors, i.e., environmental experiences to which both twins were exposed, appeared to be less important. This result was consistent for both measurement time points and thus comparable to previous research findings on other psychological constructs [26, 64]. Our results provide evidence that genetic and environmental contributions to BSI were not influenced by changing environmental conditions associated with social distancing measures. Thus, not only did we find a pattern consistent with previous research [31, 65], but we also were able to show, in answer to our second research question (Q2), that genetic and environmental contributions to BSI appeared to be fairly stable over the course of the pandemic.

Regarding the influences of the various covariates, our results were only partially consistent with previous research. In line with other research, we found that women had higher scores of BSI compared with men [12]. We found no effect for single households, which is in contrast to most previous studies (e.g., [6]). However, our sample was relatively young and included a large proportion of non-adults compared to other studies. Therefore, the characteristics of single households in our study may not be representative of the entire adult population. Because loneliness is unevenly distributed over the life course and the causes of loneliness are age-specific [66], we would also expect different effects of household composition on BSI in different age cohorts. In addition, socioeconomic indicators such as the twins' mother's education or the twins' economic position showed no effect on BSI in either lockdown. However, previous studies have already been inconsistent, finding either no association or a negative effect of socioeconomic indicators on various facets of BSI [46]. Pre-pandemic loneliness had a consistently significant effect on BSI. Thus, it may be that loneliness—along with birth cohort and gender—captures some processes relevant to the development of pandemic BSI.

With respect to our third research question (Q3), whether factors previously identified as influencing BSI were driven by genetic, shared, or non-shared environmental influences, the inclusion of the covariates mainly resulted in a decrease in the A component. This reduction in A has been linked to pre-pandemic loneliness experiences, which have mainly genetic influences on BSI. While this finding underscores the need for genetically sensitive research, it is important to acknowledge that—even after accounting for the covariates—most of the variance in our models remained unexplained (explained variance at T1: 2.8%; at T2: 1.2%). Although we were able to narrow down areas where other potentially relevant influencing factors might be expected, further research is needed to uncover the main explanatory factors for the causes of BSI. Given that much of the variance in BSI is due to genetic influences, we suspect that traits that convey genetic influences, such as personality traits, will be of primary importance at this point. While facets of personality are significantly related to the ability to cope with stressful situations, i.e., resilience (e.g., [67]), personality traits in particular have been shown to reflect genetic influences on various outcomes, including loneliness [13]. Therefore, future research should focus on the importance of personality traits as important explanatory factors to better understand who was at greater risk of being affected by BSI during the pandemic. To date, the potential influence of the twins’ personalities has been accounted for in analytical models only through the latent random variables (i.e., the variance components).

### Limitations

This study is not without limitations. First, BSI was collected in different modes in the two COVID-19 surveys (T1: online, T2: telephone, or computer-based survey). The different survey modes may affect respondents’ answers and consequently affect our results. However, because the questions used in this study did not contain sensitive information, it seems rather unlikely that response tendencies, such as social desirability, led to a significant bias in respondents’ answers. Second, participants were asked to retrospectively rate their BSI at T1, whereas their current burden was measured at T2. Retrospective assessment may be affected by recall bias, which may lead to underestimation or overestimation of the actual perceived BSI (e.g., [68]). However, in retrospective measurement, inaccuracies can be reduced by providing personal or public landmarks (e.g., [69]). This was addressed in the questionnaire for T1 by defining the first period of the pandemic to be remembered, which can be assumed to have been well recalled by the respondents because of many sudden restrictions due to the crisis. In addition, the T1 survey took place immediately after the first lockdown, so we assume that respondents had a good recall of their experience with the pandemic. Third, case numbers were lower in T1 than in T2 because only some of the respondents had participated in the first COVID-19 survey. However, in terms of sociodemographic characteristics, the two samples did not differ at the two time points (T1, T2) (Table 2). Moreover, the results presented were robust for the individuals who participated in both surveys. Fourth, due to the limited sample size, it was not possible to compute a full longitudinal model with specific environmental interactions. However, separate samples allowed us to fully utilize all available information, which increased test power and provided the most accurate estimates.

Fifth, the scale used here for BSI is not one of the commonly used scales for measuring loneliness (e.g., the UCLA-3, [45]). However, the scale we used captures what we intended to measure according to the typically used definition of loneliness, namely the subjective feeling that social relationships are perceived as insufficient or unsatisfactory [16, 19]. In line with this definition, on a semantic level, our BSI scale additionally captured the impact of loneliness on psychological distress during the pandemic, rather than just loneliness per se (as compared with, e.g., the UCLA-3). With respect to our research question, we consider this a strength of the study because the BSI scale allowed for a combined assessment of social loneliness and its associated burden as one possible aspect affecting mental health during the pandemic. Nevertheless, loneliness is a multifaceted construct (e.g., social and emotional loneliness, [70]) and we do not differentiate between the different facets of loneliness and its varying effects on mental, physical, or psychological health [71]. This should be considered when interpreting the results and attempting to draw general conclusions about loneliness based on our BSI measure during the pandemic.

Finally, the reliability of the BSI scale was only close to the recommended minimum of 0.7. Measurement error in the BSI can lead to a reduction in the estimated variance components, as the error is captured in the non-shared environmental component. However, considering all measures (Cronbach's alpha, McDonald's omega, factor analysis, and average item correlation), we conclude that the three-item scale used is adequate to answer our research question.

### Conclusion

Our results highlight the need to consider genetic and environmental influences as part of the explanation for why individuals differ in their experience of BSI over the course of the pandemic. In this context, we found a consistent pattern of non-shared environmental influences being most important, in addition to genetic influences. Although shared environmental influences did not contribute to BSI, this does not mean that apparently shared environments, such as the family environment of younger twins, have no influence on twins' experience of BSI. Rather, the results suggest that objectively shared environments do not have homogeneous effects on twins' experiences of BSI and increase their trait similarity. In other words, the environmental conditions that contribute to BSI are more likely to do so in individual ways. Our results also suggest that many covariates identified in previous studies explain only a small proportion of the observed variance. Thus, more extensive research is needed to actually uncover the key explanatory factors for the causes of BSI. Here, our study provides further clues as to where additional explanatory factors should be sought in future research. For future analyses, we recommend the use of genetically sensitive approaches, particularly those that allow testing of processes related to gene-environment interactions. For example, it is possible that individuals with a higher genetic predisposition to BSI are more likely to be found in certain environments or that certain predispositions are triggered by environmental experiences (which may be referred to as gene-environment correlation). A more precise distinction is particularly important in determining which groups of individuals are at higher risk for developing BSI.

## Supplementary Information

**Additional file 1**. Genetic and environmental contributions to the subjective burden of socialisolation during the COVID-19 pandemic.

## Data Availability

The TwinLife data are archived in the GESIS data catalogue: https://search.gesis.org/research_data/ZA6701, https://doi.org/10.4232/1.13932. Data are only released for academic research and teaching after the data depositor's written authorization.
