# Supplementary material for: Genetic and environmental contributions to the subjective burden of social isolation during the COVID-19 pandemic
Source: BMC Psychol. 2023 Apr 26;11:134. doi: 10.1186/s40359-023-01174-7 (PMC10131475; doi:10.1186/s40359-023-01174-7)
Supplement: Supplementary file 1 — Additional file 1. Genetic and environmental contributions to the subjective burden of socialisolation during the COVID-19 pandemic. [file 40359_2023_1174_MOESM1_ESM.docx]

**Additional file 1 of Genetic and environmental contributions to the subjective burden of social isolation during the COVID-19 pandemic**

**Table S1 Principal-component factor analysis: rotated factor loadings (oblique rotation)**

| Item | Factor 1 | Factor 2 | Uniqueness |
| --- | --- | --- | --- |
| burden due to… |  |  |  |
| **social isolation / loneliness** | **0.829** | -0.051 | 0.324 |
| **being separated from important people** | **0.783** | -0.034 | 0.394 |
| **lack of leisure activities** | **0.696** | -0.037 | 0.527 |
| unregulated daily routine/weekly rhythm | 0.572 | 0.213 | 0.589 |
| increased need for childcare | -0.021 | 0.950 | 0.104 |
| home schooling of children | -0.028 | 0.944 | 0.117 |
| overwork at job | 0.229 | 0.453 | 0.710 |
| restriction of civil rights | 0.507 | 0.144 | 0.699 |
| N | 3,348 |  |  |

Source: TwinLife CoV1 v6-0-0 (T1), all respondents aged >= 16

**Table S2 ACE decomposition of burden of social isolation and loneliness**

|  | **T1:**  **burden of social isolation (3-item-score)** | | | | | | **T2:**  **burden of social isolation (3-item-score)** | | | | | |
| --- | --- | --- | --- | --- | --- | --- | --- | --- | --- | --- | --- | --- |
|  | reduced model | | | full model | | | reduced model | | | full model | | |
|  |  |  |  |  |  |  |  |  |  |  |  |  |
|  | **ACE decomposition (variance components)** | | | | | | | | | | | |
|  |  |  |  |  |  |  |  |  |  |  |  |  |
| A | 0.223 |  | [-0.123,0.575] | 0.204 |  | [-0.148,0.558] | 0.246 |  | [0.054,0.437] | 0.223 |  | [0.030,0.416] |
| C | 0.125 |  | [-0.173,0.400] | 0.127 |  | [-0.171,0.404] | 0.123 |  | [-0.034,0.274] | 0.137 |  | [-0.021,0.289] |
| E | 0.652 |  | [0.543,0.775] | 0.669 |  | [0.557,0.796] | 0.631 |  | [0.567,0.701] | 0.639 |  | [0.574,0.710] |
|  |  |  |  |  |  |  |  |  |  |  |  |  |
| N | 798 |  |  | 798 |  |  | 2520 |  |  | 2520 |  |  |
| N twin pairs | 399 |  |  | 399 |  |  | 1260 |  |  | 1260 |  |  |

95% confidence intervals in brackets

Note: Reduced model adjusted for birth cohort and gender

Source: TwinLife CoV1 (T1), CoV2 (T2); Sample: cohorts 2, 3 & 4

**Table S3 Model selection**

|  | **Δ -2LL** | **p-value** | **AIC** | **BIC** |  | **Δ -2LL** | **p-value** | **AIC** | **BICs** |
| --- | --- | --- | --- | --- | --- | --- | --- | --- | --- |
|  | reduced model | | | |  | full model | | | |
|  | **T1:**  **burden of social isolation**  **(N=798)** | | | | | | | | |
| ACE |  |  | 2202.875 | 2230.798 |  |  |  | 2196.629 | 2264.441 |
| CE | 1.602 | 0.206 | 2202.477 | 2226.411 |  | 1.311 | 0.252 | 2195.939 | 2259.763 |
| **AE** | **0.707** | **0.400** | **2201.582** | **2225.516** |  | **0.739** | **0.390** | **2195.368** | **2259.191** |
|  |  |  |  |  |  |  |  |  |  |
|  | **T2:**  **burden of social isolation**  **(N=2520)** | | | | | | | | |
| ACE |  |  | 6887.090 | 6923.062 |  |  |  | 6882.944 | 6970.305 |
| CE | 6.337 | 0.012 | 6891.427 | 6922.260 |  | 5.055 | 0.025 | 6885.999 | 6968.221 |
| **AE** | **2.373** | **0.123** | **6887.463** | **6918.296** |  | **2.955** | **0.086** | **6883.899** | **6966.120** |

Source: TwinLife CoV1 (T1), CoV2 (T2); Sample: cohorts 2, 3 & 4
